# P-106. Exploration of a Desirability of Outcome Ranking (DOOR) Endpoint for Invasive Aspergillosis Using Two Registrational Trials

**DOI:** 10.1093/ofid/ofaf695.335

**Published:** 2026-01-11

**Authors:** Olufemi Ajumobi, Ursula Waack, Mark Needles, Sanjay Revankar, Daniel Rubin, Peter Kim, Vance G Fowler, Scott R Evans, Toshimitsu Hamasaki, Ramya Gopinath

**Affiliations:** Oak Ridge Institute for Science and Education, United States Department of Energy, Oak Ridge, TN, USA, Oak Ridge, Tennessee; Food and Drug Administration, Silver Spring, Maryland; FDA, Silver Spring, Maryland; Center for Drug Evaluation and Research, United States Food and Drug Administration, Silver Spring, MD, USA, Silver Spring, Maryland; FDA, Silver Spring, Maryland; US FDA, Silver Spring, Maryland; Duke University Medical Center, Durham, NC; Milken Institute School of Public Health, Rockville, MD; The George Washington University, Rockville, MD; Food and Drug Administration, Silver Spring, Maryland

## Abstract

**Background:**

By combining efficacy and safety outcomes into a single ordinal endpoint, desirability of outcome ranking (DOOR) may facilitate a holistic examination of patients’ experiences in clinical trials. We derived a syndrome-specific DOOR endpoint for invasive aspergillosis (IA) utilizing data from two registrational phase 3 clinical trials submitted to the FDA; Trial 1 compared isavuconazole to voriconazole, while Trial 2 compared posaconazole to voriconazole. Treatment duration in both trials was 6 weeks with possible extension to Day 84 [end of treatment (EOT)]; both utilized a primary endpoint of mortality at Day 42 [test of cure (TOC)]. A Data Review Committee (DRC) categorized clinical response at EOT in each trial as success (complete or partial) or failure (stable or progression).

**Methods:**

Death (entire study period), absence of clinical response (ACR; death by Day 42), infectious complications (IC), serious adverse events (SAE) and discontinuations due to treatment-related adverse events (D-TRAE) were included as components in the DOOR endpoint with a 5-level ordinal scale. We applied the resultant IA-specific DOOR to patient-level data from the two trials to estimate the probability that a patient in one treatment arm would have a more desirable outcome than a patient in the other at both TOC and EOT. Sensitivity analyses were done with ACR encompassing (1) DRC-adjudicated clinical progression or (2) DRC-adjudicated clinical progression and stability rather than mortality.

**Results:**

There were no statistically significant differences in overall DOOR probabilities between the treatment arms for Trials 1 and 2 at TOC and EOT. The DOOR probability for D-TRAE nominally significantly favored the isavuconazole arm at EOT in Trial 1. No differences between treatment arms were seen in the sensitivity analyses when DRC-adjudicated definitions of clinical failure were used as ACR.

**Conclusion:**

Though overall IA-specific DOOR probabilities between treatment arms in the two trials were similar, our work highlighted possible differences in the occurrences of D-TRAE. These results warrant further investigation and underscore the importance of key clinical data collection and consideration of improvements in the electronic case report form used in IA trials.

**Disclosures:**

Vance G. Fowler, MD, MHS, Affinergy, Janssen, Contrafect: Advisor/Consultant|AstraZeneca; EDE; Basilea: Grant/Research Support|Debiopharm, GSK; Affinium, Basilea,: Advisor/Consultant|Destiny, Amphliphi, Armata, Akagera: Advisor/Consultant|Merck; Contrafect; Karius; Janssen: Grant/Research Support|UpToDate: Royalties|Valanbio: Stock options

